# Supplementary material for: The N6-methyladenosine demethylase ALKBH5 regulates the hypoxic HBV transcriptome
Source: PLoS Pathog. 2024 Jan 16;20(1):e1011917. doi: 10.1371/journal.ppat.1011917 (PMC10817175; doi:10.1371/journal.ppat.1011917)
Supplement: Attachment — Submitted filename: HBV ALKBH5 R1 Response to reviewers 13-12-23.pdf [file ppat.1011917.s009.pdf]

NDM RESEARCH BUILDING

NUFFIELD DEPARTMENT of MEDICINE

UNIVERSITY OF OXFORD

13th December 2023

Profs Robert Kalejta and Blossom Damania

PLoS Pathogens

Re: Submission of PPATHOGENS-D-23-01343 revised manuscript: 'The N6-methyladenosine demethylase ALKBH5 regulates the hypoxic HBV transcriptome'.

Dear Prof Kalejta and Damania,

We'd like to thank you both and the external reviewers for their positive comments on our manuscript and constructive suggestions for additional experiments. We provide a point-by-point response to the reviewers comments and we believe the revisions strengthen our conclusions and our edits have improved the readability of our paper. To reflect the additional effort our co-author Peter Wing has provided in preparing the revised MS we have swapped his author listing with Aleem Siddiqui – all authors have agreed to this and we provide a signed appendix to this cover letter.

Detailed response to Reviewers Comments:

Part I – Summary *Please use this section to discuss strengths/weaknesses of study, novelty/significance, general execution and scholarship.*

Reviewer #1: The manuscript by Tsukuda et al. addresses the contribution of the N6-methyladenosine (m^6A) demethylase ALKBH5 to the hypoxia-mediated increase in HBV transcripts. The data in general are of good quality. However, some of the data and conclusions are confusing and sometimes appear to be inconsistent, and to some extent come across as a collection of potentially interesting datasets without a clearly established link/causality. This complicates the assessment if and how the data contribute to our understanding of HBV biology and the impact of hypoxia in this respect.

Response: We appreciate a request for greater clarity and to inform the reader of how hypoxic-signalling impacts the HBV life cycle. We provide a detailed response to each of the points below.

Reviewer #2: In this manuscript, the authors have investigated the mechanisms underpinning the regulation of the hepatitis B virus transcriptome in hypoxia. They conclude a role for N6-methyladenosine demethylase ALKBH5 in this response. This is a follow on from previous work from this group demonstrating a role for the hypoxia inducible factor (HIF) to activate the basal core promoter to transcribe pre-genomic RNA. Hypoxia increased viral RNA abundance in a manner associated with m^6A methyladenosine modifications. The authors went on to investigate the impact of hypoxia on RNA demethylases and found increased expression of ALKBH5 in hepatocytes in a HIF dependent manner. Silencing ALKBH5 reduced HBV pre-genomic RNA. Chronic HBV infection remains a disease of global importance lacking in curative treatment and therefore research in this area is of importance. A key strength is the work on viral transcription. The authors, however need to address the questions I have outlined in relation to the role of HIF-1 and HIF-2 in this response.

Response: We thank the reviewer for their positive comments and provide a detailed point-by-point response to the major issues below.

Part II – Major Issues: Key Experiments Required for Acceptance *Please use this section to detail the key new experiments or modifications of existing experiments that should be* ***absolutely*** *required to validate study conclusions. Generally, there should be no more than 3 such required experiments or major modifications for a "Major Revision" recommendation. If more than 3 experiments are necessary to validate the study conclusions, then you are encouraged to recommend "Reject".*

Reviewer #1: 1. A major comment is that I currently do not see the connection between different datasets and do not see what working hypothesis the authors have regarding their data. Based on the data, the way I see it: expression of the m6A demethylase ALKBH5 is increased during hypoxia, (ii) ALKBH5 then somehow

(how exactly?) prevents degradation of HIF isoforms by the proteasome, leading (iii) to an increased HIF-mediated expression of viral mRNAs. But, in this scenario, why are the m6A sites in viral RNAs important (= Figure 1 and Figure 2 of the manuscript and earlier data) as these seem to have nothing to do with point (i) to (iii) above? The authors need to design additional assays to assess and explain potential causal relationships between their findings, e.g. how does ALKBH5 prevent degradation of HIF, how and where do the m6A sites in the viral transcripts come into play – currently these seem to be separate sets of observations. This should allow them to come up with a (possibly partly hypothetical) model of what they believe is going on.

Response: We have revised our manuscript with the goal of simplifying and integrating data sets to address specific Qs on the underlying mechanisms of how hypoxia positively regulates HBV replication. The introduction now states our hypothesis: **Methylation of HBV RNAs defines their abundance under low oxygen conditions that prevail in the liver and ALKBH5 is a key regulator of this process.** Data presented in Figs.1-2 directly address this hypothesis and shows that m^6A modifications in the 5'stem-loop regulate steady-state levels of the basal core promoter (BCP) derived transcripts. A huge thanks for suggesting the Roadblock PCR technique as a more accurate method to measure RNA half-life [1] in place of the ActD pulse chase protocol used in our earlier MS. We present new data in Fig.3 using the Roadblock assay to show that hypoxia significantly increased the half-life of BCP-RNAs in wild-type HBV infections but not m^6A-null, demonstrating a role for m^6A-modification in limiting transcript stability. We are excited about the potential of this methodology for future studies on spliced RNA stability and application to other viruses. Further analysis of our long-read RNAseq data mapped BCP-RNAs with 5' m^6A modifications and this identified a role for hypoxia in the regulation of pC and pgRNA splicing – this data is presented in a new Fig.4 and Supplementary Fig.2). These data are consistent with recent studies reporting an association of m^6A levels with an alternative splicing transcriptome [2, 3] including the adenovirus RNA transcriptome [4]. Our mapping of transcriptional start sites (TSS) of HBV spliced RNAs identified an altered usage under hypoxic conditions that may relate to methylation-dependent changes in transcript stability.

To understand how ALKBH5 regulates HIF protein expression we reviewed the literature and found several reports showing that histone deacetylase 4 (HDAC4) regulates HIF-1α protein acetylation and stability [5, 6]. A recent study reported that silencing ALKBH5 increased m^6A-modification of *HDAC4* regulated HIF-1α protein expression [7] sparked our interest. We designed an experiment to assess the role of ALKBH5 to regulate *HDAC4* RNA methylation by quantitative methylated RNA immunoprecipitation (MeRIP) in ALKBH5 silenced HepG2 cells and show a significant increase in m^6A-modified *HDAC4* under our experimental conditions. These data suggest that increasing ALKBH5 expression under hypoxic conditions alters the dynamics of HDAC4 RNA methylation and HIF-1α protein stability. We include these data as new Supplementary Fig.6 along with associated text in the revised MS. We agree that our earlier cartoon depicting how ALKBH5 regulates the hypoxic HBV transcriptome was too simplistic and lacked detail. We provide a revised Fig.7 model that depicts a central role for ALKBH5 in positively regulating BCP-RNAs and in stabilizing HIF-α isoform expression, potentially via HDAC4.

2. Figure 3B: based on the Western blot, ALKBh5 protein levels seem to be only moderately increased in hypoxic conditions. Were independent replicates performed? Since this is a central point in the manuscript, the authors should quantify the apparent differences in protein levels and perform statistical analysis. This is especially important since in the Western blot shown in Supplementary Figure S3, there is no apparent increase in ALKBH5 protein levels in hypoxic (1% O2) versus 'normal' conditions (18% O2). The authors should explain and clarify these apparent conflicting results.

Response: We have performed additional kinetic experiments to quantify ALKBH5 protein expression under hypoxic conditions and these data are presented in a new Fig.5B. We show a representative blot of three independent experiments that were quantified by densitometric scanning and these data are presented adjacent to the western blot in Fig.5B. In relation to the western blot in earlier Supplementary Fig.S3 this represented a long exposure to show the differential silencing of the ALKBH5 siRNAs and so the signals were saturated. We have reviewed shorter exposure of western blots from siRNA ALKBH5 experiments under hypoxic conditions and can confirm a consistent upregulation of ALKBH5 under hypoxic conditions. We are planning to upload all raw data set to Mendely Repository and to provide a link once the paper is accepted.

3. I do not agree with the statement (lines 193 and onwards) that 'hypoxia did not alter the half-life of HBV WT pgRNA but we noted a significant reduction in m6A-null pgRNA under these conditions'. This refers to Figure 1E. In that figure, one can see that half-life of WT pgRNA goes down from 13.9h to 12.2h on average while the half-life of m6A-null pgRNA goes down from 8.3h to 6.9h on average. Hence, on average, half-life of WT pgRNA is reduced with 1.7h in hypoxic conditions, while that of m6Anull pgRNA is reduced with 1.4h in hypoxic conditions. That seems to be a very similar reduction. The standard deviation of this reduction is lower for m6Anull pgRNA compared to WT pgRNA, which likely explains the different outcome in statistical analysis. However, I very much doubt that this difference (if any) amounts to an actual biological difference. Hence, I do not agree with the statement. Additional assays would be needed to support such statement. For

example, Roadblock qPCR assays could be performed to accurately assess whether there is a difference in mRNA degradation.

Response: Thanks for recommending the Roadblock PCR technique to assess differences in HBV RNA half-life. We include new data in Fig.3 that shows hypoxia increases HBV BCP-RNA half-life and this is dependent on m^6A-modificaiton. Given the wide-ranging and possible off-target effects of ActD we are more confident to interpret this improved data set.

4. Lines 176-177, referring to Figure 1C: authors indicate that SP1 transcript levels were reduced in HBV m6Anull samples compared to WT, with a concomitant increase in SP14. This decrease/increase is not noticeable in Figure 1C – are these differences statistically significant?

Response: After further analysis of our long-read RNA-seq sequencing data and mapping TSS of BCP-derived spliced RNAs in m^6A-null and wild type infections there are no significant changes in the absolute pattern of transcripts (Supplementary Table 1).

5. Does hypoxia affect expression of any of the other relevant players in m6A biology (writer complex components, erasers, readers)?

Response: We include gene expression data in new supplementary Fig.3 that shows minimal changes in transcript levels of enzymes involved in the m^6A editing pathway.

Reviewer #2:

1. In the introduction and discussion, the authors should broaden their discussion of HIF to the well described and complex role of HIF in infection, inflammation and immunity.

Response: We have revised our MS to discuss the role of HIFs in inflammation and immunity

2. In figures 1 and 2, the authors use CA9 mRNA as a surrogate for HIF activation. The authors need to also demonstrate the temporal effects of infection, hypoxia and hydroxylase inhibitor treatment on HIF-1 and HIF-2 levels in wild type and hepatocytes by western blot to confirm its activation and compare its activation levels under the various treatments applied.

Response: We include new data in Fig.1A and Fig.2A that shows HIF-1α and HIF-2α expression by western blot in uninfected and HBV infected cells in response to low oxygen (1% oxygen) or FG-4592 treatment.

3. The authors need to confirm that hypoxic activation of ALKBH5 expression is HIF-1 or HIF-2 dependent using HIF knock down in this system. Similarly, does knockdown of HIF-1 or HIF-2 (or both) reduce alterations in viral transcription in response to hypoxia in this system. Similarly, does knockdown of HIF-1 or HIF-2 (or both) reduce alterations in viral transcription in response to hypoxia this system.

Response: Thalhammer *et al.* [8] reported the presence of two hypoxia-response elements (HREs) at -486/-482 and +265/+269 in the ALKBH5 promoter and chromatin precipitation confirmed HIF-1α bnding to this region. As both HIF-1α and HIF-2α recognize the same HRE consensus sequence, both HIF isoforms have been reported to activate ALKBH5 expression [9].To clarify this point, we have revised our MS to state that both HIF isoforms can activate ALKBH5 and cite these relevant papers. In relation to the role of HIFα isoforms in regulating HBV transcription – we previously published that both HIF-1α and HIF-2α could activate HBV transcription and we have revised the text to expand on this and cited our earlier publication [10].

Part III – Minor Issues: Editorial and Data Presentation Modifications.*Please use this section for editorial suggestions as well as relatively minor modifications of existing data that would enhance clarity.*

Reviewer #1:

1. Line 99: only METTL3 functions as a methyltransferase. METTL14 serves as a catalytically inactive allosteric activator of METTL3.

Response: We apologise for this mis-citation and have revised the sentence accordingly.

2. Line 100-101: suggest to rephrase to 'This complex typically methylates adenosine residues within the consensus DRACH…': methylation does not exclusively occur in this consensus sequence, so add 'typically'.

Response: Thank you for the comment. As the reviewer suggested, we have modified text to read "This complex typically methylates adenosine residues within the consensus DRACH (D=A, G or U; R=G or A; H=A,

C or U) motif which is often located near stop codons, 3' untranslated regions and internal exons in mRNAs [11, 12]"

3. Line 264: I presume the authors mean that beta-actin transcripts were unchanged (rather than HIF-1alpha)?

Response: We apologize for any confusion. We wanted to explain that we observed HIF protein reduction in ALKBH5 silenced cells without changing HIF-1α mRNA levels. We have revised this sentence to read: "*Cells transfected with siALKBH5 showed a significant reduction in the expression of both HIF-α protein levels and associated NDRG1 expression without changing HIF-1α mRNA level*."

4. Line 279: I presume the authors mean that the data are 'consistent with a role for the demethylase in preventing destabilization of both isoforms' (rather than a role in destabilizing the isoforms as currently indicated)?

Response: Thank you for pointing out this error - we have corrected the sentence to read: "*CHX treatment inhibited HIF-1α and HIF-2α expression, and their degradation was accelerated in the absence of ALKBH5, consistent with a role for the demethylase in stabilizing both isoforms.*"

Reviewer #2:

1. Please avoid the use of the phrase "there was a trend albeit non-significant" (Page 8).

Response: We have revised the sentence to read: "*HIF-1α mRNA is methylated in HepG2 cells although there was no significant difference between hypoxic or normoxic conditions.*"

We look forward to favorable review and to see our work published in PLoS Pathoges.

With best wishes,

Jane A McKeating on behalf of all authors

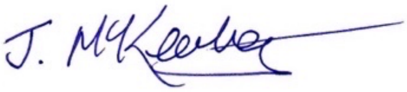

References

1. Watson MJ, Thoreen CC. Measuring mRNA Decay with Roadblock-qPCR. Curr Protoc. 2022;2(1):e344. doi: 10.1002/cpz1.344. PubMed PMID: 35041257; PubMed Central PMCID: PMCPMC8830782.

2. Zhou KI, Shi H, Lyu R, Wylder AC, Matuszek Z, Pan JN, et al. Regulation of Co-transcriptional Pre-mRNA Splicing by m(6)A through the Low-Complexity Protein hnRNPG. Mol Cell. 2019;76(1):70-81 e9. Epub 20190821. doi: 10.1016/j.molcel.2019.07.005. PubMed PMID: 31445886; PubMed Central PMCID: PMCPMC6778029.

3. Tan B, Zeng J, Meng F, Wang S, Xiao L, Zhao X, et al. Comprehensive analysis of pre-mRNA alternative splicing regulated by m6A methylation in pig oxidative and glycolytic skeletal muscles. BMC Genomics. 2022;23(1):804. Epub 20221206. doi: 10.1186/s12864-022-09043-0. PubMed PMID: 36474138; PubMed Central PMCID: PMCPMC9724443.

4. Price AM, Hayer KE, McIntyre ABR, Gokhale NS, Abebe JS, Della Fera AN, et al. Direct RNA sequencing reveals m(6)A modifications on adenovirus RNA are necessary for efficient splicing. Nat Commun. 2020;11(1):6016. Epub 20201126. doi: 10.1038/s41467-020-19787-6. PubMed PMID: 33243990; PubMed Central PMCID: PMCPMC7691994.

5. Zhang X, Qi Z, Yin H, Yang G. Interaction between p53 and Ras signaling controls cisplatin resistance via HDAC4- and HIF-1alpha-mediated regulation of apoptosis and autophagy. Theranostics. 2019;9(4):1096-114. Epub 20190130. doi: 10.7150/thno.29673. PubMed PMID: 30867818; PubMed Central PMCID: PMCPMC6401400.

6. Zhang Y, Ren YJ, Guo LC, Ji C, Hu J, Zhang HH, et al. Nucleus accumbens-associated protein-1 promotes glycolysis and survival of hypoxic tumor cells via the HDAC4-HIF-1alpha axis. Oncogene. 2017;36(29):4171-81. Epub 20170320. doi: 10.1038/onc.2017.51. PubMed PMID: 28319066; PubMed Central PMCID: PMCPMC5537617.

7. Liu X, Feng M, Hao X, Gao Z, Wu Z, Wang Y, et al. m6A methylation regulates hypoxia-induced pancreatic cancer glycolytic metabolism through ALKBH5-HDAC4-HIF1alpha positive feedback loop. Oncogene. 2023;42(25):2047-60. Epub 20230506. doi: 10.1038/s41388-023-02704-8. PubMed PMID: 37149664; PubMed Central PMCID: PMCPMC10275754.

8. Thalhammer A, Bencokova Z, Poole R, Loenarz C, Adam J, O'Flaherty L, et al. Human AlkB homologue 5 is a nuclear 2-oxoglutarate dependent oxygenase and a direct target of hypoxia-inducible factor 1alpha (HIF-1alpha). PLoS One. 2011;6(1):e16210. Epub 2011/01/26. doi: 10.1371/journal.pone.0016210. PubMed PMID: 21264265; PubMed Central PMCID: PMCPMC3021549.

9. Zhang C, Samanta D, Lu H, Bullen JW, Zhang H, Chen I, et al. Hypoxia induces the breast cancer stem cell phenotype by HIF-dependent and ALKBH5-mediated m(6)A-demethylation of NANOG mRNA. Proc Natl Acad Sci U S A. 2016;113(14):E2047-56. Epub 2016/03/24. doi: 10.1073/pnas.1602883113. PubMed PMID: 27001847; PubMed Central PMCID: PMCPMC4833258.

10. Wing PAC, Liu PJ, Harris JM, Magri A, Michler T, Zhuang X, et al. Hypoxia inducible factors regulate hepatitis B virus replication by activating the basal core promoter. J Hepatol. 2021;75(1):64-73. Epub 2021/02/01. doi: 10.1016/j.jhep.2020.12.034. PubMed PMID: 33516779; PubMed Central PMCID: PMCPMC8214165.

11. Dominissini D, Moshitch-Moshkovitz S, Schwartz S, Salmon-Divon M, Ungar L, Osenberg S, et al. Topology of the human and mouse m6A RNA methylomes revealed by m6A-seq. Nature. 2012;485(7397):201-6. Epub 2012/05/12. doi: 10.1038/nature11112. PubMed PMID: 22575960.

12. Meyer KD, Saletore Y, Zumbo P, Elemento O, Mason CE, Jaffrey SR. Comprehensive analysis of mRNA methylation reveals enrichment in 3' UTRs and near stop codons. Cell. 2012;149(7):1635-46. Epub 2012/05/23. doi: 10.1016/j.cell.2012.05.003. PubMed PMID: 22608085; PubMed Central PMCID: PMCPMC3383396.

NDM RESEARCH BUILDING

NUFFIELD DEPARTMENT of MEDICINE

UNIVERSITY OF OXFORD

12th December 2023

Editors at PLoS Pathogens

Manuscript Number: PPATHOGENS-D-23-01343 revised MS: 'The N6-methyladenosine demethylase ALKBH5 regulates the hypoxic HBV transcriptome'.

New Authorship listing: Senko Tsukuda, James M Harris, Andrea Magri, Peter Balfe, Aleem Siddiqui, Peter AC Wing and Jane A McKeating.

We approvide the new authorship listing where Aleem Siddiqui and Peter Wing have swapped places in the R1 revised MS.

Best wishes,

Senko Tsukuda 九十田千子

James Harris J. HARRIS

Andrea Magri

Peter Balfe

Peter Wing

Jane A McKeating

Aleem Siddiqui – please see screen shot of email

Dear Jane
By all means, do as you see appropriate.
Cheers Akeem

Sent from my iPad

On Dec 6, 2023, at 6:29 AM, Jane McKeating <jane.mckeating@ndm.ox.ac.uk> wrote:

Dear Aleem

Hope all is well.

We're getting ready to submit the revised ALKBH5 MS to PLoS Pathogens next week.
Senko has some very nice new data and we'll forward a copy of the revised MS early next.
I want to ask if you'd be willing to change co-author position as Peter as has helped Senko with the revised draft.

New Authorship would be:

Senko Tsukuda[1]*, James M Harris[1], Andrea Magri[1], Peter Balfe[1], Aleem Siddiqui[3], Peter AC Wing[2], and Jane A McKeating[1,2]*.
